# The predictive value of circulating inflammatory and immune biomarkers for stroke-associated pneumonia following endovascular therapy in patients with acute anterior circulation large vessel occlusion infarction: a prospective cohort study

**DOI:** 10.3389/fneur.2026.1764204

**Published:** 2026-02-26

**Authors:** Yongsen Lin, Ting Lin, Biyu Wu, Zhuquan Hong, Zhihua Zhu, Xiaorong Zhang, Quanlong Hong, Pingzhen Lin

**Affiliations:** 1 Department of Neurology, Quanzhou First Hospital Affiliated to Fujian Medical University, Quanzhou, China; 2 Department of Neurology, Zhangzhou Affiliated Hospital of Fujian Medical University, Zhangzhou, China; 3 Nursing Department, Quanzhou First Hospital Affiliated to Fujian Medical University, Quanzhou, China

**Keywords:** acute anterior circulation large vessel occlusion infarction, endovascular therapy, immune, inflammatory, stroke-associated pneumonia

## Abstract

**Objective:**

To investigate the changes in circulating biomarkers of patients with acute anterior circulation large vessel occlusive cerebral infarction (ACLVO) following endovascular therapy (EVT), and to explore their potential utility as early predictors for the development of stroke-associated pneumonia (SAP).

**Methods:**

Peripheral blood samples were collected from ACLVO patients on days 1, 3, and 7 following EVT. Samples were analyzed to detect monocyte human leukocyte antigen-DR (mHLA-DR) expression level, along with plasma levels of interleukin-6 (IL-6), C-reactive protein (CRP), and procalcitonin (PCT).

**Results:**

Multivariate binary logistic regression analysis adjusted for clinical confounders identified decreased mHLA DR expression at day 1 and dysphagia as independent SAP predictors (*P* < 0.001). Compared to the non-SAP group, the SAP group showed significantly lower expression of mHLA-DR on days 1, 3, and 7 (*P* < 0.001), plasma IL-6 and CRP levels were significantly higher on days 3 and 7 (*P* < 0.05), as was the plasma PCT level on day 7 (*P* < 0.05). Infarct volume was significantly larger in the SAP group (*P* < 0.001). In predictive modeling, expression of mHLA-DR on days 1 alone yielded an *AUC* of 0.914 (optimal cutoff: 55.75%). Decision curve analysis showed that expression of mHLA-DR on days 1 offered greater net benefit than other clinical scores, and that combining it with the A2DS2 score provided a superior, stable net benefit for SAP prediction.

**Conclusion:**

In this exploratory study, our findings suggest that mHLA-DR expression levels may indicate some predictive value for SAP development after EVT in ACLVO patients, with the predictive performance appearing enhanced when combined with the A2DS2 score. Dysphagia and a large infarct volume emerged as potential risk factors for SAP. Conversely, within the context of our cohort, plasma levels of IL-6, CRP, and PCT did not demonstrate clear utility for the early prediction of SAP.

## 1 Introduction

Acute ischemic stroke (AIS) is a major global threat to human health and has emerged as the leading cause of death and disability among residents in both urban and rural areas of China (1). Data from 2020 indicated that the prevalence of stroke in Chinese adults aged 40 years and older reached 2.6% (2). Around 40% of AIS cases are caused by large vessel occlusion (LVO), and these patients often experience severe cerebral infarction, associated with high rates of disability and mortality (3). In recent years, endovascular therapy (EVT) has become the cornerstone of treatment for LVO patients, significantly improving their prognostic outcomes (4). However, patients with LVO typically present with large infarct volumes, severe paralysis, and a high risk of complications, such as pneumonia (5). These factors pose challenges to clinical management and emphasize the critical importance of timely treatment of complications for ensuring favorable AIS patient prognosis.

Stroke-associated pneumonia (SAP) is a common complication of AIS, occurring in 5%−26% of patients and most frequently within the first 48 h after stroke onset (6, 7). SAP not only significantly prolongs hospitalization and increases healthcare costs but also contributing to higher short- and long-term mortality among AIS patients (8, 9). Studies indicate that the incidence of SAP is notably higher in patients with LVO undergoing EVT than in those not receiving EVT (5). Therefore, identifying risk factors for SAP in LVO patients undergoing EVT is crucial for developing effective prevention and treatment strategies. Although various early clinical prediction models for SAP have been proposed, most rely on scale-based assessments, which are inherently limited by rater experience and patient cooperation, NIHSS score in particular is susceptible to these factors (10, 11), and its accuracy may be further compromised in patients with severe strokes such as LVO, who typically present with NIHSS scores >10 (12). These limitations underscore the need for objective and robust biomarkers to enhance SAP risk prediction in this patient population.

Several risk factors for SAP have been identified in existing studies, including advanced age, atrial fibrillation, dysphagia, stroke severity, and immunosuppression (13). Of these factors, immunosuppression has been identified as an independent risk factor for SAP (14). After AIS, the release of immune-inflammatory mediators protects brain tissue by suppressing excessive inflammation. However, this response concurrently induces a state of immunosuppression, known as stroke-induced immunodepression syndrome (SIDS), which is characterized by reduced lymphocyte counts, impaired T helper cell function, and dysregulated monocyte activation. These changes can emerge within 12–24 h post-stroke, predisposing AIS patients to an increased risk of SAP (15, 16). Notably, the use of prophylactic antibiotics has not demonstrated benefits in reducing the incidence of SAP, improving long-term functional outcomes, or decreasing mortality (17). As a result, recent researches has shifted focus toward evaluating and modulating the immunosuppressed state as a novel SAP prevention strategy (18). Thus, the early identification of high-risk SAP patients via inflammatory and immune biomarkers, followed by timely immunomodulatory or targeted antibiotic therapy, may therefore offers a promising approach to improving the prognosis of AIS patients (19).

Although multiple inflammatory and immune factors have been demonstrated to be closely associated with the development of SAP, no single factor or combination of factors has been fully translated into clinically practical predictors (15). Previous studies have shown that plasma levels of interleukin-6 (IL-6), C-reactive protein (CRP), and procalcitonin (PCT) are significantly elevated in patients with SAP during the early phase of stroke, suggesting their potential utility as biomarkers for predicting SAP (15, 20). The monocyte system is critical for immune regulation in the context of AIS development, and perturbations in its function may act as potential biomarkers to reflect altered immune status in patients with AIS (21). In the monocyte lineage, the expression of human leukocyte antigen-DR (mHLA-DR) on the surface of monocytes plays a central role in the immune response by presenting both exogenous and endogenous antigenic peptides to CD4^+^ T cells. Thus, mHLA-DR is widely used as a key marker to evaluate monocyte activity (22). Previous evidence further supports that decreased mHLA-DR expression is a significant predictor of SAP, with stroke patients exhibiting low mHLA-DR levels facing a substantially increased risk of pneumonia (14, 23). However, it remains unclear whether these biomarkers can effectively predict the risk of SAP in LVO patients undergoing EVT and warrants further investigation.

Therefore, this study employs a prospective design to examine the dynamic changes in mHLA-DR expression and plasma levels of IL-6, CRP, and PCT in patients with acute anterior circulation large vessel occlusion (ACLVO) following EVT, further seeks to assess the potential predictive value of these markers for SAP, with the objective of contributing to earlier identification of high-risk patients.

## 2 Materials and methods

### 2.1 Research participants

This prospective cohort study enrolled patients with ACLVO who were admitted to Quanzhou First Hospital Affiliated to Fujian Medical University between January and December 2024. The inclusion criteria were as follows: (1) aged over 18 years; (2) diagnosis of AIS, as confirmed by imaging and attributable to acute occlusion of the internal carotid artery, the M1 or M2 segments of the middle cerebral artery, or the A1 segment of the anterior cerebral artery; (3) admitted within 12 h of symptom onset; and (4) having given consent to undergo EVT. Patients were excluded if they met any of the following criteria: (1) intracranial hemorrhage, as confirmed by imaging; (2) pre-stroke modified Rankin Scale (mRS) score ≥ 2; (3) contraindication to EVT due to allergy to iodinated contrast agents; (4) current infection, antibiotic prophylaxis at admission, or history of infection within 30 days prior to onset; (5) requirement for mechanical ventilation at symptom onset or anticipated need for mechanical ventilation; (6) history of hematological diseases, malignancy, or ongoing immunosuppressive therapy; (7) severe hepatic or renal dysfunction; (8) presence of brain tumors with mass effect on neuroimaging; or (9) Expected inability to complete follow-up.

This study was approved by the ethics committee of Quanzhou First Hospital Affiliated to Fujian Medical University (Approval No.: 2023K089) and registered with chictr.org.cn (Registration No.: ChiCTR2500111972). The informed consent was obtained from all enrolled patients.

### 2.2 Criteria for SAP diagnosis

SAP was diagnosed according to the Chinese Expert Consensus on the Diagnosis and Treatment of Stroke-Associated Pneumonia (24). SAP was defined as pneumonia occurring within 7 days after stroke onset and meeting at least one of the following criteria: (1) fever (body temperature ≥38 °C) without other identifiable cause; (2) leukopenia ( ≤ 4 × 10^9^/L) or leukocytosis (≥10 × 10^9^/L); or (3) age ≥70 years with altered mental status of unclear etiology. In addition, at least two of the following clinical features were required: (1) new purulent sputum, change in sputum characteristics, increased respiratory secretions, or increased suction frequency within 24 h; (2) new onset or worsening cough, dyspnea, or tachypnea (respiratory rate > 25 breaths/min); (3) pulmonary auscultatory findings such as rales, crackles, or bronchial breath sounds; or (4) impaired gas exchange. Furthermore, chest imaging had to demonstrate at least one of the following: new or progressive infiltrates, consolidation, or ground-glass opacities.

### 2.3 Baseline data

In this study, we collected the following baseline data: (1) demographic characteristics, including age and sex; (2) past medical history and social history, including hypertension, diabetes, atrial fibrillation, chronic obstructive pulmonary disease (COPD), smoking status, and alcohol consumption; and (3) stroke-related clinical characteristics, including thrombolytic therapy, dysphagia, responsible vessel, EVT procedural steps, degree of recanalization (mTICI), infarct location, infarct volume, haemorrhagic transformation, National Institutes of Health Stroke Scale (NIHSS) score at admission, A2DS2 score, ISAN score, and TOAST aetiological classification.

### 2.4 Follow-up visits

A total of 137 participants were enrolled, of whom 84 were finally included in the study according to the inclusion and exclusion criteria. All enrolled patients underwent EVT. Fasting venous blood was collected into four glass tubes containing ethylenediaminetetraacetic acid (3 mL into each tube) at 07:00 a.m. on days 1, 3, and 7 after treatment. The samples were subsequently analyzed using various detection techniques: flow cytometry to determine mHLA-DR expression levels, enzyme-linked immunosorbent assay to determine plasma IL-6 levels, immunofluorescence chromatography to determine plasma PCT levels and immunoturbidimetry to determine plasma CRP levels (14, 25–27). Concurrently, dynamic disease assessments were conducted at baseline (prior to EVT) and on days 3 and 7 post-treatment. Neurological deficits were evaluated using the NIHSS score, and cranial computed tomography (CT) scans were performed to monitor hemorrhagic transformation and assess changes in infarct volume. Additionally, patients were monitored throughout the follow-up period for the occurrence of SAP, which was diagnosed based on predefined criteria. Finally, dynamic changes in inflammatory and immune parameters in the blood were compared between the SAP and non-SAP groups during follow-up.

The primary outcome was the incidence of SAP. The secondary outcomes included: longitudinal changes in expression levels of mHLA-DR, plasma IL-6, CRP, and PCT at various postoperative time points, along with their predictive value for SAP; the predictive utility of the NIHSS score for SAP; and the relationship between cerebral infarction volume and SAP occurrence.

### 2.5 Statistical analysis

Statistical analyses were performed using SPSS 25.0 (SPSS Inc., Chicago, IL, USA) and R (version 4.4.1; R Foundation for Statistical Computing, Vienna, Austria). The primary and secondary outcomes were subjected to the per-protocol set (PPS) analysis. Normality of data distribution was assessed using Q-Q plots, frequency histograms, and the Kolmogorov-Smirnov test. Continuous variables that followed a normal distribution were expressed as mean ± standard deviation (SD), while those that did not follow a normal distribution were summarized as median [interquartile range, IQR]. Categorical variables were presented as number (percentage). For intergroup comparisons of baseline characteristics, Mann-Whitney U test was applied for non-normally distributed continuous variables, while independent samples t-test was used for normally distributed continuous variables. Categorical variables were compared using the chi-square test or Fisher's exact test. Variables with a *P*-value < 0.05 in univariate analyses were entered into a multivariable binary logistic regression model to identify independent risk factors for SAP. Sensitivity analysis using multiple linear regression was conducted to evaluate potential bias resulting from conceptual overlap between SAP diagnostic criteria and the NIHSS score. Trends over time within and between groups were assessed using repeated-measures analysis of variance.

The predictive performance of predictors for SAP was evaluated using receiver operating characteristic (ROC) curve analysis, with the area under the curve (AUC) serving as the quantitative measure of discrimination. To further validate the robustness and reproducibility of the prediction model, an internal validation was performed using the non-parametric bootstrap method with 1000 replications. Decision-curve analysis (DCA) was performed in R to assess the clinical utility and incremental value of mHLA-DR in predicting SAP. Net benefit was calculated over a threshold probability range of 0–0.8. The following seven clinical strategies were compared: “treat all,” “treat none,” NIHSS score alone, A2DS2 score alone, ISAN score alone, and mHLA-DR combined separately with each of these three scores. A higher net benefit indicates greater clinical usefulness of the corresponding strategy at a given risk threshold. All statistical tests were two-sided, and a *P*-value < 0.05 was considered statistically significant.

## 3 Results

### 3.1 Research participants

**Figure 1:**
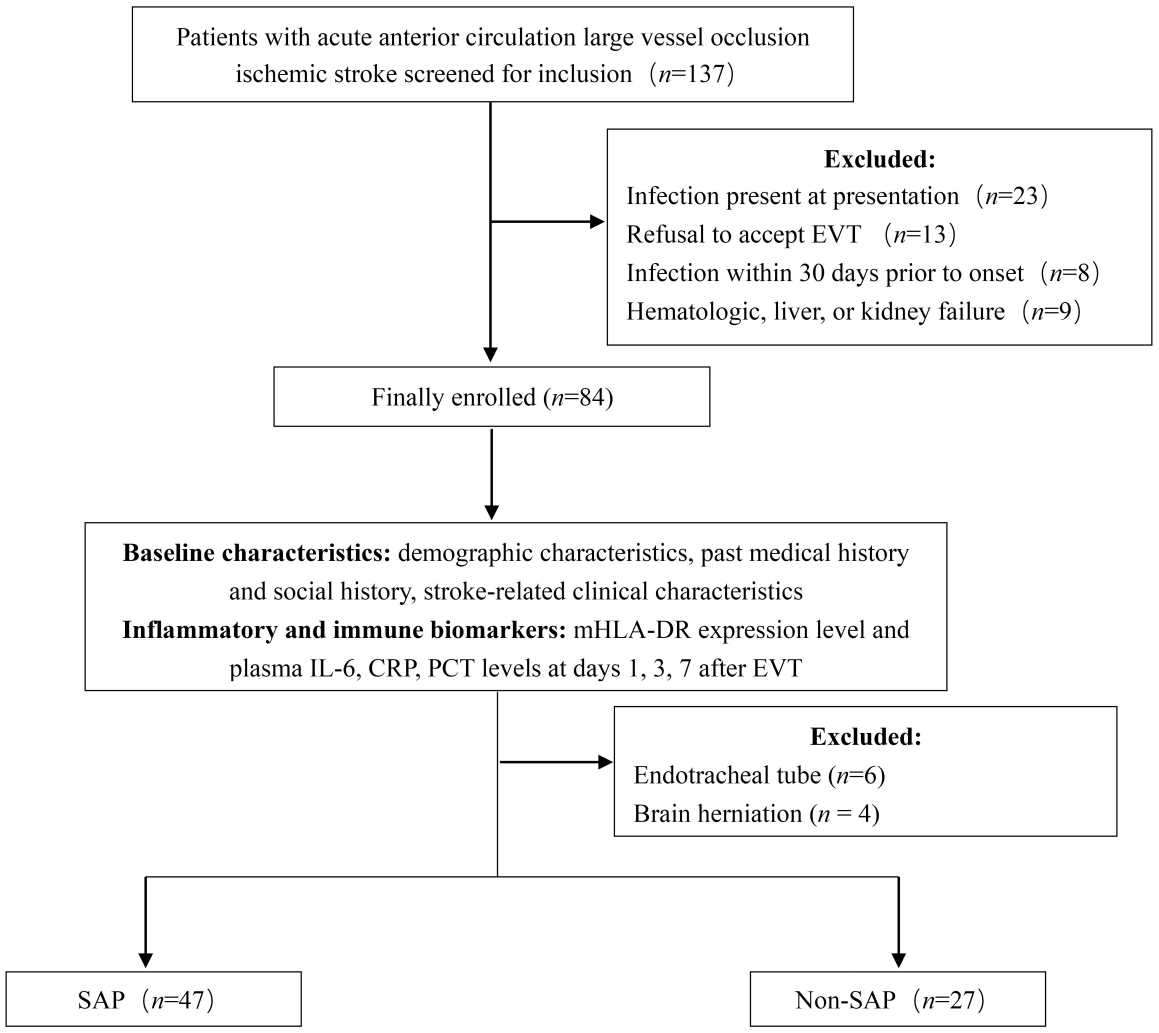
Patient follow-up flow chart. EVT, endovascular treatment; SAP, stroke-associated pneumonia; mHLA-DR, monocyte human leukocyte antigen-DR; IL-6, Interleukin-6; CRP, C-reactive protein; PCT, Procalcitonin.

The follow-up flow chart for enrolled patients is shown in Figure 1. A total of 137 patients were initially included in the study. Of these, 53 were excluded based on the exclusion criteria, leaving 84 patients enrolled. During the 7-day follow-up period, six patients withdrew due to endotracheal intubation, and four patients were automatically discharged due to brain herniation. Consequently, 74 patients ultimately completed the study.

### 3.2 Baseline characteristics of participants

The baseline characteristics of the study participants are summarized in Table 1. Among the 74 patients who completed follow-up, 47 (63.51%) developed SAP at a median of 2.36 days, while 27 did not. Significant differences were observed between the SAP and non-SAP groups in sex, dysphagia, infarct volume, responsible vessels, EVT technique, A2DS2 score, ISAN score, and baseline NIHSS score (*P* < 0.05). Compared with the non-SAP group, mHLA-DR expression on day 1 post-EVT was markedly lower in SAP patients (*P* < 0.001). Based on these differences, a multivariate binary logistic regression was performed to identify predictors of SAP. Results demonstrated that dysphagia and decreased levels of mHLA-DR expression on day 1 post-EVT were independent risk factors for SAP (Supplementary Table 1).

**Table 1:** Baseline characteristics of participants.

**Item**	**SAP (*n* = 47)**	**Non-SAP (*n* = 27)**	***t/u/χ^2^***	***P***
Time to SAP (days)	2.36 (1.97–2.76)	*N/A*	*N/A*	*N/A*
Gender (Male/Female)	32/15	12/15	3.976	**0.046**
Age (years)	64 (52–69)	67 (51–73)	568	0.455
Hypertension (*n*, %)	20 (42.55%)	9 (33.33%)	0.612	0.434
Diabetes (*n*, %)	22 (46.81%)	10 (37.04%)	0.667	0.414
Atrial fibrillation (*n*, %)	19 (40.43%)	9 (33.33%)	0.367	0.545
COPD (*n*, %)	12 (25.53%)	5 (18.52%)	0.477	0.490
Smoking (*n*, %)	18 (38.20%)	6 (22.22%)	2.022	0.155
Alcohol (*n*, %)	26 (55.32%)	10 (37.04%)	2.294	0.130
Thrombolysis (*n*, %)	16 (34.04%)	8 (29.63%)	0.152	0.696
Dysphagia	40 (85.11%)	9 (33.33%)	20.548	**< 0.001**
Infarct location			1.795	0.180
Right	25	10		
Left	22	17		
TOAST			1.554	0.460
Large artery atherosclerosis	24	19		
Cardiogenic	18	8		
Arterial dissection	5	2		
Infarct volume (ml)	138.14 ± 73.91	44.08 ± 30.30	−6.301	**< 0.001**
Responsible vessel			25.079	**0.001**
R-ICA	3	1		
R-MCA	10	7		
R-ACA	0	2		
R-ICA+R-MCA	11	0		
L-ICA	3	0		
L-MCA	10	11		
L-ACA	1	4		
L-ICA+L-MCA	9	2		
EVT technique			10.421	**0.015**
ADAPT	15	2		
SWIM	22	22		
ReWiSed CARe	4	2		
BASIS	6	1		
mTICI			3.203	0.202
2b	2	0		
2c	5	1		
3	40	26		
HT (*n*, %)	15 (31.91%)	6 (22.22%)	0.793	0.373
NIHSS (points) *Baseline*	18 (14–19)	8 (5-11)	92	**< 0.001**
A2DS2	7.09 ± 1.54	4.19 ±2.34	−5.764	**< 0.001**
ISAN	9.77 ± 2.87	6.59 ± 3.20	−4.261	**< 0.001**
mHLA-DR (%) Day 1	48.79 ± 8.66	63.50 ± 5.67	7.898	**< 0.001**
IL-6 (pg/ml) Day 1	4.02 ± 1.89	3.99 ± 1.64	−0.082	0.935
CRP (mg/L) Day 1	5.41 (2.78–7.73)	4.59 (2.89–6.85)	553	0.360
PCT (ng/ml) Day 1	0.04 (0.04–0.058)	0.04 (0.04–0.064)	575.5	0.464

Data are shown as mean ± standard deviation or M (median), IQR (interquartile range) or n (%).

SAP, stroke-associated pneumonia; COPD, chronic obstructive pulmonary disease; TOAST, Trial of ORG 10172 in Acute Stroke Treatment; ICA, internal carotid artery; MCA, middle cerebral artery; ACA, anterior cerebral artery; EVT, Endovascular Therapy; ADAPT, a direct aspiration first pass technique; SWIM, Solitaire stent with intracranial support catheter for mechanical thrombectomy; ReWiSed CARe, retriever wire supported carotid artery revascularization; BASIS, balloon angioplasty with the distal protection of stent retriever; mTICI, modified Treatment in Cerebral Ischemia; HT, Hemorrhagic Transformation; NIHSS, National Institutes of Health Stroke Scale; A2DS2, Age, Atrial fibrillation, Dysphagia, Sex, Stroke Severity; ISAN, Prestroke Independence, Sex, Age, National Institutes of Health Stroke Scale; mHLA-DR, monocyte human leukocyte antigen-DR; IL-6, Interleukin-6; CRP, C-reactive protein; PCT, Procalcitonin.

Bold values indicate statistically significant results.

To assess potential bias due to conceptual overlap between criteria for SAP diagnosis and NIHSS score, a sensitivity analysis was conducted using multiple linear regression. After adjusting for age and baseline disturbance of consciousness, the association between SAP and NIHSS score remained statistically significant (*P* < 0.001), although the effect size was attenuated, as indicated by a 28% decrease in the β coefficient from 6.445 to 4.641 (Supplementary Table 2).

### 3.3 Comparison of inflammatory and immune biomarkers in blood between SAP and non-SAP groups

For both the SAP group and the non-SAP group, repeated measures analysis of variance was conducted for mHLA-DR expression levels and plasma IL-6, CRP, and PCT levels at each time point. The results showed that, there were significant main effect of both time and grouping for above biomarkers (*P* < 0.05), with plasma IL-6 and CRP levels also showing a significant time × group interaction (*P* < 0.05), while no significant interaction was observed for mHLA-DR expression levels and plasma PCT levels (Table 2).

**Table 2:** The results of repeated measures analysis of variance between two group and Cohen's *d* with 95 % CI of the two groups at each time point.

**Outcome**	Time		Group		Time × group interaction		Cohen's ***d*** effect size (95 %CI)		
	***F***	***P***	***F***	***P***	***F***	***P***	**Baseline or Day 1**	**Day 3**	**Day 7**
mHLA-DR (%)	21.505	**< 0.001**	45.449	**< 0.001**	1.162	0.312	**−1.907 (−2.469**, **−1.336)***^***a***^*	**−1.510 (−2.039**, **−0.972)**	**−1.148 (−1.654**, **−0.636)**
Plasma IL-6 level	17.737	**< 0.001**	14.596	**< 0.001**	7.616	**0.001**	0.020 (**–**0.454, 0.493)*^***a***^*	**0.983 (0.481, 1.480)**	**0.561 (0.077, 1.041)**
Plasma CRP level	4.961	**0.013**	12.743	**0.001**	4.374	**0.021**	0.246 (**–**0.229, 0.721)*^***a***^*	**0.847 (0.351, 1.337)**	**0.622 (0.136, 1.104)**
Plasma PCT level	4.128	**0.034**	4.262	**0.043**	2.977	0.074	**–**0.036 (**–**0.510, 0.437) *^***a***^*	0.423 (**–**0.057, 0.900)	**0.564 (0.080, 1.044)**
NIHSS score	167.211	**< 0.001**	75.513	**< 0.001**	3.940	**0.028**	**2.069 (1.507, 2.675)** *^***b***^*	**1.933 (1.359, 2.497)**	**1.754 (1.197, 2.303)**

Bold values indicate statistically significant results. a, Day 1; b, Baseline.

SAP, stroke-associated pneumonia; mHLA-DR, monocyte human leukocyte antigen-DR; IL-6, Interleukin-6; CRP, C-reactive protein; PCT, Procalcitonin; NIHSS, National Institutes of Health Stroke Scale.

**Figure 2:**
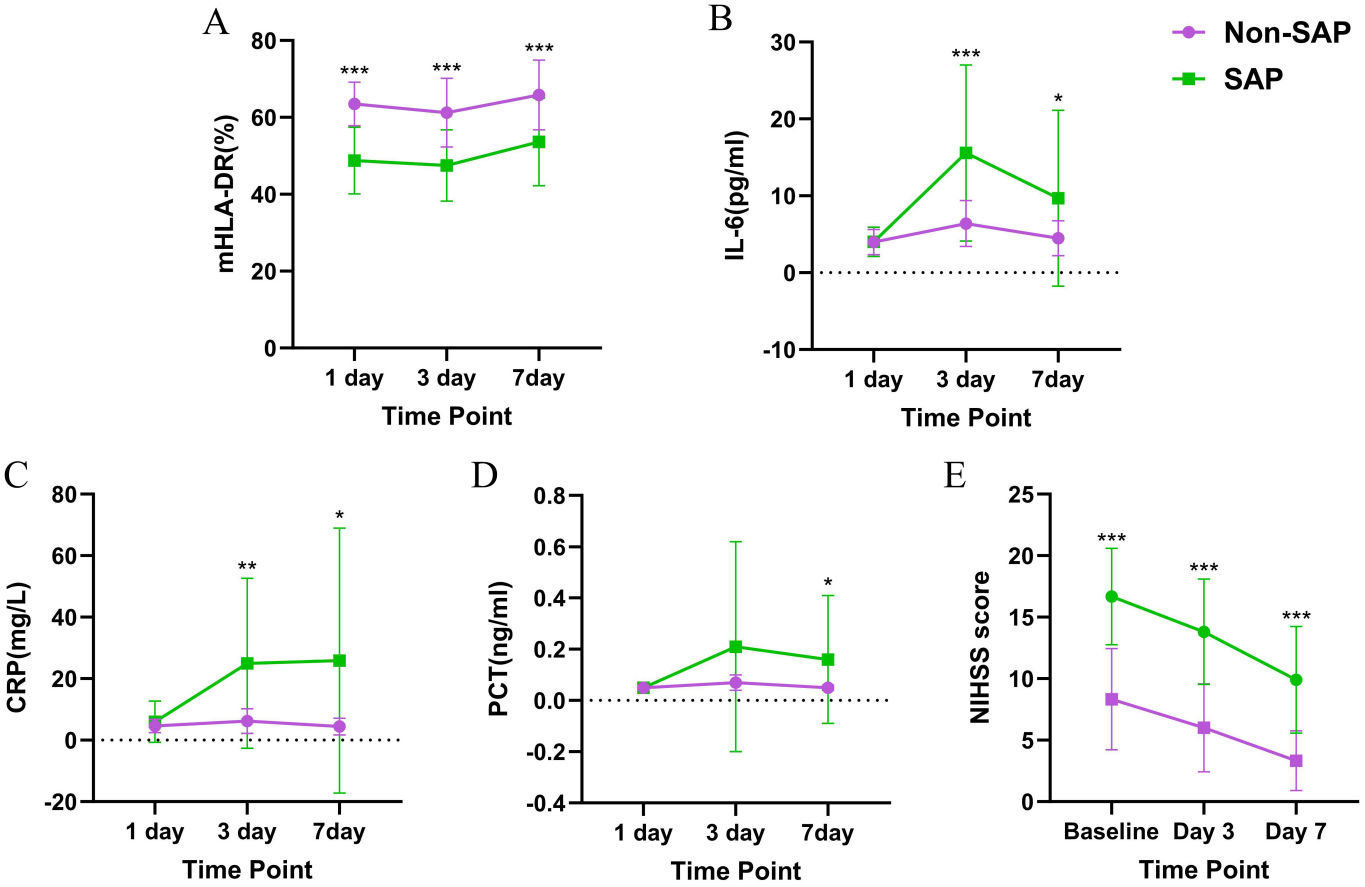
Comparison of **(A)** mHLA-DR expression, **(B)** plasma IL-6 levels, **(C)** plasma CRP levels, **(D)** plasma PCT levels, and **(E)** NIHSS scores between the SAP group and the non-SAP group. SAP, stroke-associated pneumonia; mHLA-DR, monocyte human leukocyte antigen-DR; IL-6, interleukin-6; CRP, C-reactive protein; PCT, procalcitonin; NIHSS, National Institutes of Health Stroke Scale. *p < 0.05, **p < 0.01, and ***p < 0.001.

Trends of mHLA-DR expression levels and plasma IL-6, CRP, and PCT levels at each time point in the two groups and Cohen's *d* effect size compared between groups are shown in Table 2, Figure 2, and Supplementary Table 3. In the SAP group, the mHLA-DR expression levels on days 1, 3, and 7 after treatment were lower than those in the non-SAP group (*P* < 0.001). On day 3 after treatment, the plasma IL-6 and CRP levels in the SAP group were higher than those in the non-SAP group (*P* < 0.01). By day 7 after treatment, the plasma IL-6, CRP, and PCT levels in the SAP group were higher than those in the non-SAP group (*P* < 0.05).

### 3.4 Comparison of NIHSS score between SAP and non-SAP groups

For both the SAP group and the non-SAP group, repeated measures analysis of variance was conducted for NIHSS score at each time point (Table 2, Figure 2, and Supplementary Table 3). The results showed that for NIHSS score, there were significant time and group main effects between the two groups, and there was a significant time × group interaction (*P* < 0.05). Further analysis of the NIHSS score trends at each time point, along with Cohen's *d* effect sizes, revealed that NIHSS scores in the SAP group were significantly higher than those in the non-SAP group at baseline, day 3 post-treatment, and day 7 post-treatment (*P* < 0.001).

### 3.5 Predictive value of mHLA-DR expression for SAP

**Figure 3:**
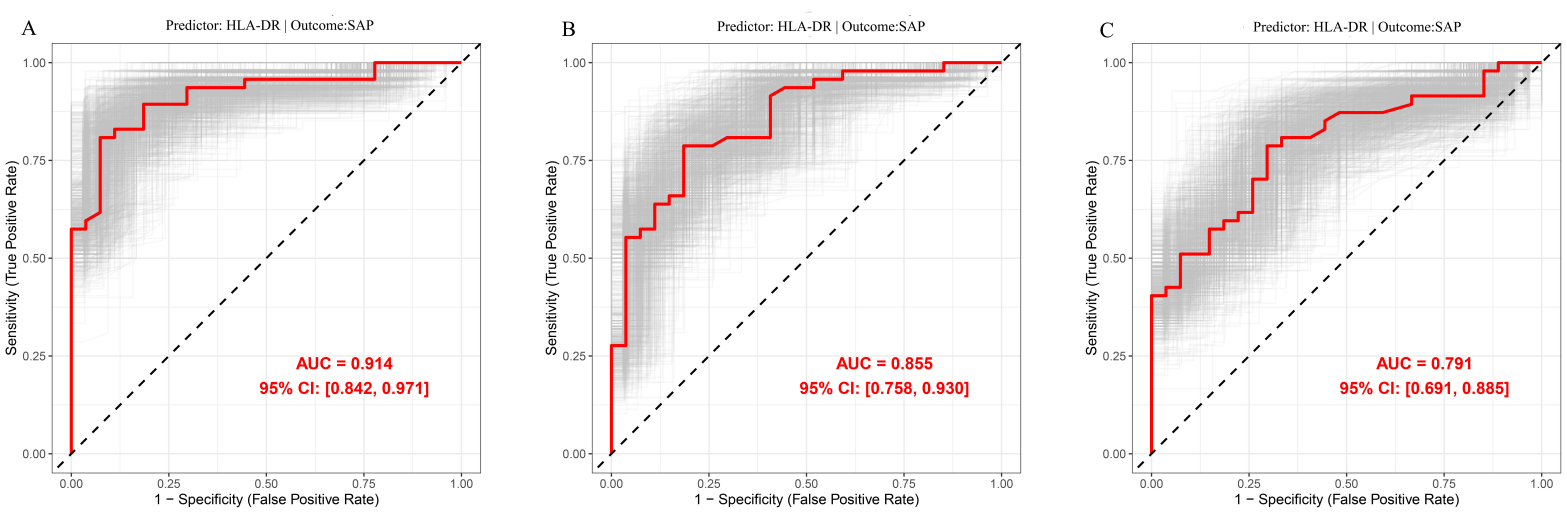
ROC curve analysis with 1000 bootstrap replicates for mHLA-DR expression in predicting SAP. **(A)** Predictive value of mHLA-DR expression for SAP at day 1. **(B)** Predictive value of mHLA-DR expression for SAP at day 3. **(C)** Predictive value of mHLA-DR expression for SAP at day 7. SAP, stroke-associated pneumonia; mHLA-DR, monocyte human leukocyte antigen-DR.

ROC analysis was used to identify the optimal mHLA-DR expression cut-off values for predicting SAP in patients with ACLVO who underwent EVT, and the results were internally validated by the nonparametric Bootstrap method (1000 repetitions). On day 1 after EVT, a cut-off value of 55.75% yielded an AUC of 0.914, with 92.6% sensitivity and 80.9% specificity. On day 3, a cut-off value of 55.15% yielded an AUC of 0.855, with 81.5% sensitivity and 78.7% specificity. By day 7, the optimal cut-off increased to 62.90%, resulting in an AUC of 0.791, with 70.4% sensitivity and 78.7% specificity (Figure 3).

### 3.6 Decision curve analysis of predictive models for SAP

**Figure 4:**
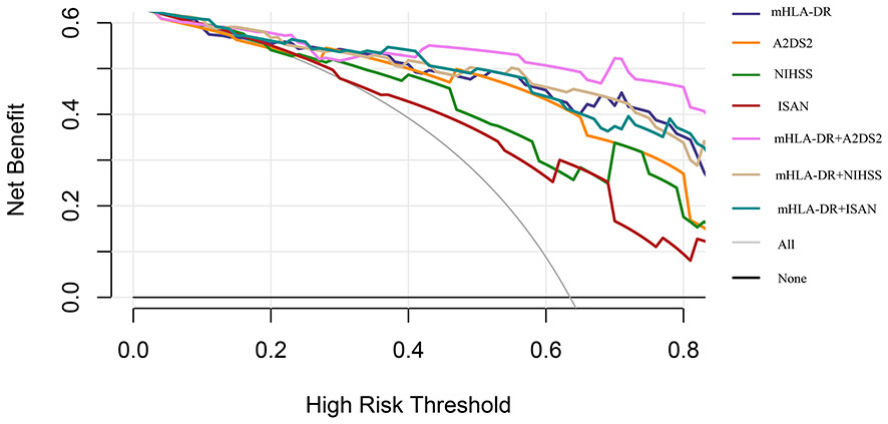
Decision curve analysis of predictive models for SAP. SAP, stroke-associated pneumonia; A2DS2, Age, Atrial fibrillation, Dysphagia, Sex, Stroke Severity; ISAN, Prestroke Independence, Sex, Age, National Institutes of Health Stroke Scale; mHLA-DR, monocyte human leukocyte antigen-DR.

Decision curve analysis (DCA) was performed to evaluate the clinical net benefit of mHLA-DR, both alone and in combination with established clinical scores (A2DS2, NIHSS, and ISAN), for predicting adverse outcomes in patients (Figure 4). The net benefit of each predictive model (mHLA-DR, NIHSS, A2DS2, and ISAN) consistently exceeded both the “no intervention” and “all intervention” strategies, indicating their potential utility in clinical decision-making. Among single-variable models, mHLA-DR demonstrated the highest net benefit. In addition, among combination models, mHLA-DR + A2DS2 yielded the greatest net benefit across the entire threshold range. Although the net benefit of all models declined as the risk threshold increased, the mHLA-DR + A2DS2 combination exhibited a more gradual decrease, suggesting better stability and clinical applicability.

## 4 Discussion

Patients with ACLVO who underwent EVT were enrolled in this prospective cohort study, and several key findings were observed. First, patients who developed SAP exhibited persistently lower levels of mHLA-DR expression throughout the follow-up period compared to patients who did not develop SAP. In addition, plasma levels of IL-6 and CRP were significantly elevated on days 3 and 7 after stroke onset, while PCT levels also increased markedly by day 7. Second, imaging analyses revealed that patients with SAP had significantly larger cerebral infarct volumes than those without SAP. Finally, in terms of predictive performance, dysphagia and mHLA-DR expression level were identified as independent predictors of SAP in ACLVO patients receiving EVT. Moreover, DCA analysis demonstrated that, as a single predictor, mHLA-DR achieved greater net benefit than other clinical prediction models for SAP. Moreover, mHLA-DR combination with the A2DS2 score demonstrated greater net benefit and stability.

Currently, mHLA-DR expression levels are widely used to predict the risk of infection following AIS (15). Previous studies have shown that reduced mHLA-DR expression on day 1 after AIS onset is an independent predictor of SAP (14). In this study, focusing on ACLVO patients undergoing EVT, we observed that mHLA-DR expression levels were significantly lower in SAP patients compared to non-SAP patients starting from the first postoperative day. Multivariable binary logistic regression analysis further confirmed mHLA-DR expression as an independent predictor of SAP. ROC curve analysis showed that an mHLA-DR expression level below 55.75% on the first day after SAP onset had good predictive value for SAP. Decreased mHLA-DR expression in AIS patients has been reported to predict SAP and can occur as early as 3 h after onset, often preceding functional changes in monocytes, thus serving as an early indicator of monocyte dysfunction (14, 23, 28, 29). These findings are consistent with the results of our study. In summary, for ACLVO patients treated with EVT, early reduction in mHLA-DR expression is a reliable predictor of SAP.

The persistence of low mHLA-DR expression levels suggests that immunosuppression is being maintained (30). In this study, the dynamic monitoring of mHLA-DR levels over a period of 7 days in patients with ACLVO who were receiving EVT revealed that those with SAP had consistently lower expression levels throughout the follow-up period compared to those without SAP. ROC curve analysis showed that mHLA-DR < 55.15% on day 3 and < 62.90% on day 7 after onset had a good predictive value for the development of SAP. Furthermore, patients in the SAP group exhibited significantly larger cerebral infarct volumes than those in the non-SAP group. Previous studies have similarly reported persistently low mHLA-DR expression in AIS patients who develop SAP, with greater infarct volumes correlating with more pronounced mHLA-DR reduction (23, 31, 32). The decrease in mHLA-DR expression levels in peripheral blood of SAP patients can last for 1–2 weeks, and the degree of decrease is closely related to the prognosis of patients with acute stroke (23, 31, 32). These findings suggests that monocyte dysfunction is one of the key factors predisposing to secondary infection in AIS patients, while the expansion of infarct size may further aggravate immune dysfunction (32). Continuous immunosuppression is prevalent in the acute phase of stroke, and this pathophysiological feature may be an important cause of increased susceptibility to SAP, also indicating that the immune function status of patients is closely related to the occurrence of SAP (14). Therefore, targeted intervention for the immunosuppressive mechanism in the acute phase of stroke may provide new strategies for the prevention and treatment of SAP and inform future drug development (33).

In this study, the AUC of mHLA-DR for predicting SAP reached 0.914, demonstrating strong discriminatory ability. However, such high predictive accuracy is uncommon in real-world clinical research and warrants cautious interpretation. This outcome may be attributable to factors including the single-center design, high homogeneity of the study population, stringent inclusion and exclusion criteria, as well as standardized detection methods and outcome definitions. It should also be noted that the limited sample size increases the risk of model overfitting, and the absence of external validation further restricts the generalizability of the findings (34, 35). Therefore, these results should be regarded as a promising preliminary signal rather than evidence supporting immediate clinical application. Future validation through large-scale, multicenter, and external cohorts is necessary to confirm the true predictive utility of mHLA-DR.

Previous studies have indicated that IL-6 is a strong predictor of SAP in stroke patients, it may help assess infection risk early in the disease course—within hours—with levels correlating with patient mortality (20, 36). In addition, plasma CRP and PCT are also considered potential predictive markers of SAP and significant increases in their levels are seen within 24–48 h after stroke (37). However, the present study found no statistically significant differences in plasma levels of IL-6, CRP, or PCT between SAP and non-SAP patients in the early phase. These biomarkers did not show significant divergence until day 3 or day 7. In our interpretation, this finding does not diminish the value of these biomarkers but may instead reflect a distinct post-stroke immune-inflammatory response profile in the setting of EVT.

First, the median time to SAP development in this cohort was 2.36 days, whereas systemic inflammatory responses after AIS generally peak between days 3 and 5 (33). Early blood sampling, such as within 24 h post-EVT, may therefore have captured a pre-inflammatory state before SAP became clinically evident. The differences observed after day 3 likely reflect a pronounced inflammatory response triggered by SAP itself (14), suggesting that the predictive time window for traditional inflammatory markers may need to be redefined for patients with ACLVO treated with EVT.

Second, EVT may modulate early inflammation through recanalization-related immunomodulation. Successful recanalization is known to attenuate systemic inflammation by reducing brain tissue necrosis, thereby improving outcomes (38, 39). In this study, all ACLVO patients underwent EVT, and those who developed SAP had significantly larger infarct volumes than non-SAP patients, supporting the concept that greater infarct burden exacerbates immunosuppression, and SAP risk (32). These findings suggest that inflammatory and immune parameters in both groups may be influenced by EVT. Successful recanalization likely limits infarct size, partially mitigating post-stroke immunosuppression, and systemic inflammation (40).

Finally, reverse causality must be considered. More severe baseline infarct core or neurological deficits may directly increase SAP risk and subsequent inflammatory responses, rather than early markers driving SAP development—a perspective consistent with the present findings. Therefore, the limited early predictive performance of IL-6, CRP, and PCT may partly stem from their collinearity with infarct volume as the underlying etiology (41). After EVT rapidly alters disease progression, the independent predictive utility of these markers may be diminished. In summary, for EVT-treated ACLVO patients, IL-6, CRP, and PCT appear to have limited value in the early prediction of SAP, and may be more suitable as indicators for monitoring inflammation after SAP onset. Further research is needed to clarify the dynamic evolution and mechanisms of peripheral inflammatory markers before and after EVT in AIS patients.

Our findings indicate that patients with SAP had significantly higher A2DS2, ISAN, and NIHSS scores than those without SAP, and consistently higher NIHSS scores during the first 7 days post-stroke, consistent with prior studies (42). It is important to note that scale assessment is susceptible to assessor experience and patient cooperation and is particularly evident in patients with severe stroke (10, 11). We therefore used DCA to compare the predictive utility of the objective immune marker mHLA-DR with that of clinical scales. DCA revealed that mHLA-DR alone offered a greater net benefit than any single scale, indicating its potential to reduce subjective assessment bias as an objective biomarker. This is consistent with previous evidence that blood-based inflammatory markers are more accurate than clinical scales in predicting SAP (43). Moreover, the model combining mHLA-DR and the A2DS2 score yielded the highest net clinical benefit and was the most stable across risk thresholds. These findings indicate that integrating objective immune markers with clinical scales enhances prediction robustness and clinical utility for SAP (36). However, this single-center study had a limited sample size, and the clinical applicability of the combined model requires further validation in larger, multicenter prospective cohorts.

This study confirms that the combined mHLA-DR-based model demonstrates good predictive performance and clinical net benefit for SAP. However, its clinical translation requires consideration of practical implementation factors. Currently, mHLA-DR testing by flow cytometry is available in most tertiary hospitals in China and has a turnaround time of 2–3 h, comparable to conventional inflammatory markers and suitable for early stroke risk stratification (44). Although mHLA-DR testing costs more than CRP or PCT (approximately ¥200–300 per sample), targeted prevention guided by mHLA-DR risk stratification, such as early antibiotics or enhanced airway care, can yield substantial savings, as one SAP episode increases healthcare costs by roughly ¥6000–19000 (45). In addition, mHLA-DR specifically reflects stroke-induced immunosuppression rather than systemic nonspecific inflammation (22), offering a clearer pathophysiological basis for infection prediction and demonstrating good clinical applicability.

## 5 Limitations

This study has several limitations. (1) The primary limitations of this study include its single-center design, limited sample size, and absence of external validation. These factors may restrict the model's generalizability, and external validation through multicenter studies is needed before clinical application. (2) The elevation of inflammatory-immune markers observed on day 1 in patients who were later diagnosed with SAP suggest that SAP may be initiated within the first day after stroke onset. This implies that SAP may be a trigger for immunosuppression rather than merely a consequence of it. Future studies should include ultra-early immune marker assessments within hours of stroke onset. (3) This study collected blood samples only after EVT, lacking preoperative baselines. Consequently, it remains unclear whether biomarker changes resulted from the stroke itself or the EVT procedure. Future research should systematically collect samples before, immediately after, and at multiple time points following EVT to better assess inflammatory-immune dynamics and their association with SAP.

## 6 Conclusion

In summary, this preliminary exploratory study indicates that early monitoring of mHLA-DR levels following EVT may help predict post-stroke pneumonia, particularly when integrated with the A2DS2 model. Changes in mHLA-DR occurred earlier than those in inflammatory markers such as IL-6, CRP, and PCT, suggesting its potential role as an early warning indicator. However, given the small sample size, single-center design, lack of external validation, risk of overfitting, and undefined cut-off values, these results remain hypothesis-generating and are not yet suitable for clinical use. Future multicenter, large-sample, prospective external validation studies are needed to confirm the predictive utility of mHLA-DR and to establish clinically robust, generalizable cut-off criteria, and monitoring strategies.

## Data Availability

The raw data supporting the conclusions of this article will be made available by the authors, without undue reservation.
