# Supplementary material for: The predictive value of circulating inflammatory and immune biomarkers for stroke-associated pneumonia following endovascular therapy in patients with acute anterior circulation large vessel occlusion infarction: a prospective cohort study
Source: Front Neurol. 2026 Feb 26;17:1764204. doi: 10.3389/fneur.2026.1764204 (PMC12979128; doi:10.3389/fneur.2026.1764204)
Supplement: Supplementary file 1 [file Table_1.docx]

**Supplemental** **Table 1. Multivariate binary logistic regression analysis of independent risk factors for SAP**

| **Variables** | ***B*** | ***SE*** | ***Wald*** $\chi^{2}$ | ***P*** | ***OR*** | ***95%CI（OR）*** |
| --- | --- | --- | --- | --- | --- | --- |
| Gender | -1.110 | 0.890 | 1.556 | 0.212 | 0.330 | 0.058, 1.885 |
| Infarct Volume | 0.015 | 0.017 | 0.759 | 0.384 | 1.015 | 0.982, 1.049 |
| Dysphagia | 2.056 | 0.939 | 4.798 | **0.028^*^** | 7.815 | 1.242, 49.187 |
| mHLA-DR | -0.168 | 0.085 | 3.946 | **0.047^*^** | 0.845 | 0.716, 0.998 |
| Responsible Vessel | 0.206 | 0.214 | 0.922 | 0.337 | 1.229 | 0.807, 1.870 |
| EVT Technique | 0.328 | 0.576 | 0.323 | 0.570 | 1.388 | 0.448, 4.294 |
| NIHSS score | 0.068 | 0.100 | 0.466 | 0.495 | 1.071 | 0.880, 1.303 |

Note: ^**^*p* < 0.01;

Abbreviations: *SAP*, Stroke-associated pneumonia; *EVT*, Endovascular Therapy; *SE,* Standard Error; *95% CI*, 95% Bias Corrected Confidence Interval.

**Supplemental Table 2. Multiple linear regression analysis between diagnostic criteria of SAP and NIHSS total score**

| **Variable** | **Unadjusted Model** | | **Adjust Mode^#^** | |
| --- | --- | --- | --- | --- |
|  | *B（95% CI）* | *P* | *B（95% CI）* | *P* |
| SAP | 6.455 (4.132, 8.777) | **＜0.001^***^** | 4.641 (3.112, 6.169) | **＜0.001^***^** |
| Age(year) |  |  | -0.017 (-0.072, 0.039) | 0.557 |
| mental status |  |  | 7.942 (6.371, 9.512) | **＜0.001^***^** |
| R² | 0.299 | | 0.721 | |

Note: ^***^*p* < 0.001; # Adjusted model controlled for both age and baseline disturbance of mental status.

Abbreviations: *SAP*, Stroke-associated pneumonia.

**Supplementary Table 3. Changes of blood inflammation-immune markers and NIHSS score at each time point in SAP patients and Non-SAP patients**

| Item | Time point | SAP (n=47) | Non-SAP (n=27) | *F* | *P* |
| --- | --- | --- | --- | --- | --- |
| mHLA-DR | Day 1 | 48.79±8.66 | 63.50±5.67 | 62.374 | **＜0.001** |
|  | Day 3 | 47.48±9.26 | 61.25±8.92 | 39.096 | **＜0.001** |
|  | Day 7 | 53.62±11.41*^ab^* | 65.82±9.07*^b^* | 22.617 | **＜0.001** |
| IL-6 | Day 1 | 4.02±1.89 | 3.99±1.64 | 0.007 | 0.935 |
|  | Day 3 | 15.57±11.45*^a^* | 6.39±2.98 *^a^* | 16.582 | **＜0.001** |
|  | Day 7 | 9.67±11.43*^ab^* | 4.49±2.26 *^b^* | 5.392 | **0.023** |
| CRP | Day 1 | 5.98±6.70 | 4.62±2.19 | 1.041 | 0.311 |
|  | Day 3 | 24.97±27.62*^a^* | 6.17±4.00 | 12.294 | **0.001** |
|  | Day 7 | 25.87±43.05*^a^* | 4.43±2.70 | 6.644 | **0.012** |
| PCT | Day 1 | 0.05±0.02 | 0.05±0.02 | 0.023 | 0.880 |
|  | Day 3 | 0.21±0.41*^a^* | 0.07±0.03 *^a^* | 3.064 | 0.084 |
|  | Day 7 | 0.16±0.25 *^ab^* | 0.05±0.01*^b^* | 5.451 | **0.022** |
| NIHSS score | Baseline | 16.68±3.91 | 8.33±4.11 | 75.323 | **＜0.001** |
|  | Day 3 | 13.81±4.28*^c^* | 6.00±3.57 *^c^* | 64.056 | **＜0.001** |
|  | Day 7 | 9.91±4.33*^cb^* | 3.33±2.42*^cb^* | 52.763 | **＜0.001** |

Note: *a,* significant difference compared with Day 1 (P < 0.05); *b,* significant difference compared with Day 3 (P < 0.05); *c,* significant difference compared with Baseline (P < 0.05).

Abbreviations: *SAP,* Stroke-associated pneumonia; *mHLA-DR,* monocyte human leukocyte antigen-DR; *IL-6,* Interleukin-6; *CRP,* C-reactive protein; *PCT,* Procalcitonin.
